# Association Between Anterior Hip Capsule Thickening and Sagittal Pelvic Alignment Among Patients With Developmental Dysplasia of the Hip

**DOI:** 10.7759/cureus.54370

**Published:** 2024-02-17

**Authors:** Koji Yoshikawa, Tatsuya Tamaki, Tetsuya Kimura, Yuji Matsumoto, Ryunosuke Endo, Eiki Tsushima

**Affiliations:** 1 Physiotherapy, Naka Orthopedic Kyoto Saiin Rehabilitation Clinic, Kyoto, JPN; 2 Health Sciences, Hirosaki University, Hirosaki, JPN; 3 Orthopedic Surgery, Naka Orthopedic Kyoto Kitano Main Institution, Kyoto, JPN; 4 Orthopedic Surgery, Naka Orthopedic Kyoto Saiin Rehabilitation Clinic, Kyoto, JPN; 5 Physiotherapy, Naka Orthopedic Kyoto Kitano Main Institution, Kyoto, JPN; 6 Physiotherapy, Kensei Hospital, Hirosaki, JPN

**Keywords:** hip capsular thickness, ce angle, sharp angle, sacral slope, anterior pelvic tilt, pelvic incidence, caliper, total hip arthroplasty (tha), hip capsule

## Abstract

Introduction: The pathogenesis and pathology of secondary osteoarthritis (OA) of the hip, which is mainly due to developmental dysplasia of the hip (DDH), in Japan are obscure. There are some reports on the thickening of the hip capsule, but the relationship between the thickness of the hip capsule and the pelvic alignment due to hip deformity is not well known. This research investigated whether the capsular thickness of female DDH patients was related to pelvic alignment.

Methods: This single-center cross-sectional study included female patients aged 50-79 years (n=13) who had undergone primary total hip arthroplasty (THA) due to secondary hip OA with a background of DDH. The part of the hip capsule including the iliofemoral ligament was resected and measured directly with a digital caliper. The Sharp angle, center-edge (CE) angle, sacral slope (SS), pelvic tilt (PT), pelvic incidence (PI), and lumbar lordosis angle (LLA) were measured with an X-ray image to investigate the relationship between the capsular thickness and the pelvic posture.

Results: Pearson's correlation coefficient showed a negative correlation between hip capsular thickness and Sharp angle (r=-0.57, p>0.05). No significant correlation was found between the thickness of the hip capsule and the sagittal X-ray parameters including SS, PT, PI, LLA, and CE angle in the coronal plane.

Conclusion: The thickness of the hip capsule is moderately associated with the Sharp angle on the coronal plane. The results of this study suggest that the thickness of the joint capsule does not necessarily relate to the degenerative process among patients with DDH and the process can be complex to apply two-dimensional postural indices for the explanation.

## Introduction

The prevalence of osteoarthritis (OA) of the hip diagnosed by X-ray in Japan was reported to be 1-4.3%, with a higher prevalence in women (2-7.5%) than in men (0-2%) [1,2]. The frequency of secondary hip OA is estimated to be dominant based on a study that found the frequency of primary hip dysplasia in Japan to be 0.65% [3]. Among them, the frequency of secondary hip OA due to developmental dysplasia of the hip (DDH) is estimated to be high compared with other countries [4], though the epidemiology in Japan is obscured. The most frequent age of the first occurrence of OA of the hip in Japan is in the 50s (58±14 years) among women [5]. Pain and decreased range of motion (ROM) with hip arthritis limited activities of daily living (ADL) and quality of life. However, ADL after the operation of total hip arthroplasty (THA) has improved significantly [6]. It can contribute to maintaining the traditional Japanese lifestyle represented by sitting on the floor [7]. However, the pathogenesis and pathophysiology of secondary hip arthritis caused by DDH are still not well known.

The hip joint is stable posteriorly due to its ball-and-socket joint geometry supported by the acetabular labrum and intra-articular and extra-articular ligaments [8]. On the other hand, anterior stability comparatively relies on soft tissue such as the iliopsoas muscle to ensure joint mobility [8]. Patients with hip OA have limited ROM due to irreversible degeneration of the acetabulum, femur, and labrum with or without femoral head ligament rupture [9]. In addition, thickening of the articular capsule and iliocapsularis muscle in front of the femoral head is observed, which is considered a physical response to the joint instability [9-11]. However, the validation of the thickening process of the hip capsule has been limited to anatomical studies using cadaver dissection [11,12], a joint arthroscopic study [13], and imaging studies by MRI [14-17].

The restriction of ROM in dysplastic hips has been shown to affect whole-body posture [18]. A part of the disease concept called "hip-spine syndrome," which associates the reduction of hip ROM with hip degeneration, results in compensatory lumbar extension. Further, restricted hip ROM can be one of the causes of low back pain [19]. Especially for the typical dysplastic hips in the sagittal plane, the pelvis tilts forward to compensate for the acetabular coverage, and the lordosis of the lumbar spine increases to reduce hip symptoms [19]. In the coronal plane, an increase in the Sharp angle is associated with an increase in the pelvic anteversion angle [20]. In general, the pelvic anteversion angle is less likely to change in the older population once it has increased due to hip degeneration [21]. These findings may indicate that irreversible postural changes can occur to compensate for the decreased hip function by the progression of hip OA. Our hypothesis is that pelvic alignment and anterior hip capsule thickness may be related due to postural changes associated with joint degeneration in patients with DDH.

However, to the best of our knowledge, there have been no studies that have examined the relationship between pelvic alignment and the thickness of the anterior hip capsule, by the use of direct measurement in patients with DDH. The results of this study can contribute to a deeper understanding of the pathogenesis of hip OA and the postural compensation among DDH patients. The purposes of this study were to directly measure the thickness of the hip capsule with a caliper during THA in patients with DDH and to determine whether the thickness of the anterior hip capsule is related to the degree of pelvic alignment and the curve of the lumbar spine.

## Materials and methods

This was a single-center, cross-sectional study conducted at Naka Orthopedic Kyoto Kitano Main Institution in Kyoto, Japan. The subjects were women aged 50-79 years who had primary THA for secondary hip OA as a result of DDH. The reason why male was excluded in the present study was that DDH is more common in females in our population [1,2,22] and the hip joint and pelvis are morphologically different between genders [23]. All the subjects agreed to the consent form before participation (n=13). The patients were excluded if they had primary hip arthritis, spinal or knee diseases, psychiatric disorders that may affect posture such as depression, or paralysis after cerebrovascular disease which can affect the posture. The research in this paper was conducted with the approval of the Ethics Committee of the Graduate School of Health Sciences at Hirosaki University (approval number: 2023-002).

Hip capsular measurement

**Figure 1:**
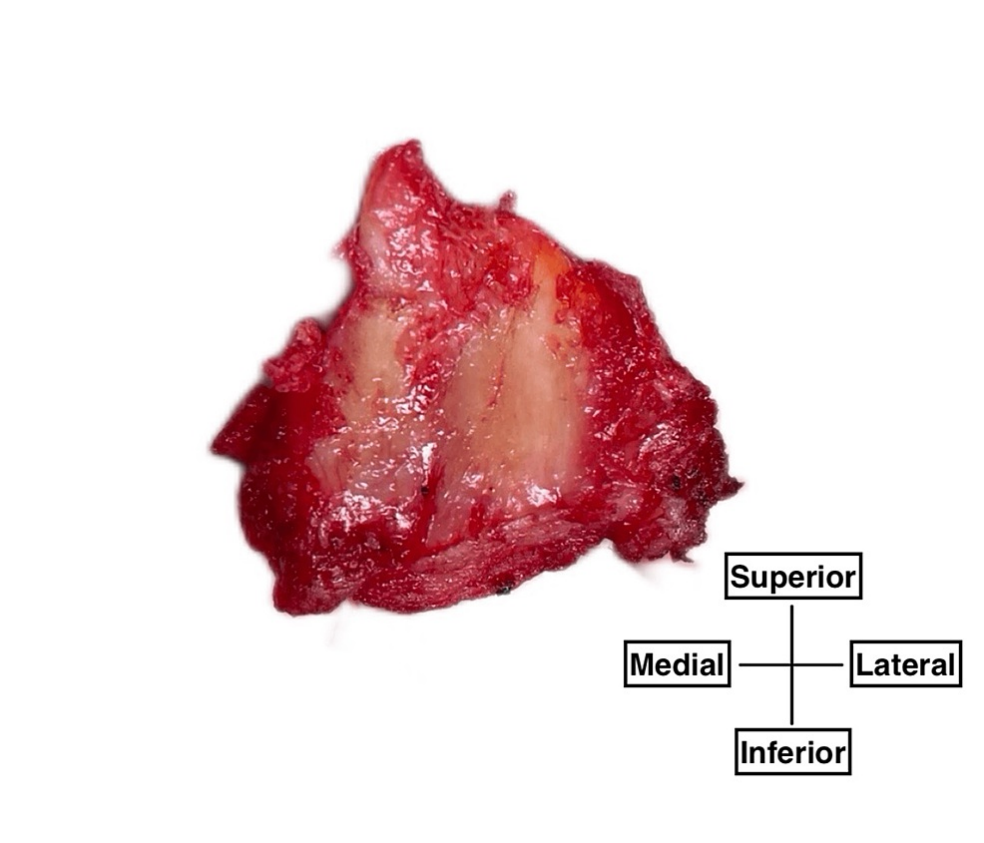
Record example of the removed hip capsule viewing from the posterior side of the specimen The white, longitudinal fibers running in the center of the image were measured.

For the measurement of hip capsule thickness, the hip capsule was measured directly with a digital caliper (Mefine, measurement accuracy: 0.01 mm) during the direct anterior THA. The anatomical location was in the anterior lateral region in front of the femoral head. From the center to the medial part of the surgical site, the lateral fiber of the iliofemoral ligament is located. The most medial side of the surgical site may partially include the medial fiber of the iliofemoral ligament. The lateral side of the surgical site is estimated as a thin part of the anterior hip capsule [11,24]. A ligated suture was marked on the capsule under the anterior inferior iliac spine (AIIS) inside the patient's surgical wound (6-10 cm long near the tensor fascia latae muscle). The capsule including the iliofemoral ligament was incised with an electrocautery scalpel where the femoral head and femoral neck are located posterior to the hip capsule. The anterior hip capsule and femoral head were extracted as medical waste. Adipose tissue was removed under direct view from the excised hip capsule to expose the joint capsular ligaments, and the specimen was photographed with the superior side up and the inferior side down, with the medial and lateral sides indicated, as well as the anterior and posterior sides (Figure 1). All the surgical process was performed by two co-authors (TT and TK).

**Figure 2:**
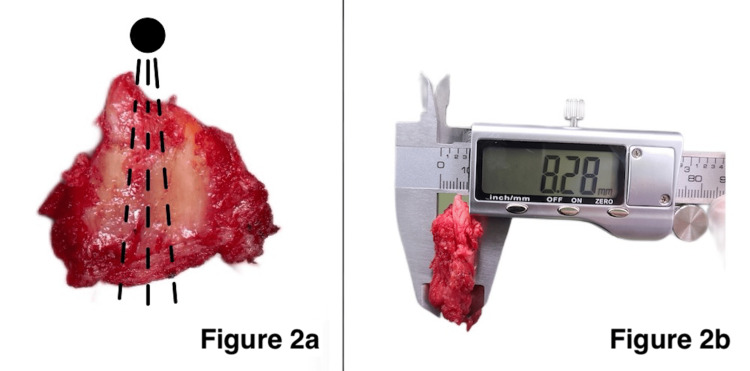
a: Location of measurement (black dashed line): AIIS location (black circle). b: Measurement by a digital caliper AIIS: anterior inferior iliac spine

A digital caliper was used to measure the thickness of the hip capsule. The measurement site was the line between the AIIS and the intertrochanteric line on the anterior surface of the femoral head, which is the area considered to be thickest [11,24]. Regarding the measurement of the capsule, the specimen was inserted deep into the caliper jaws and was measured three times with different angles within the estimated thickened fibers of the anterior hip capsule. Photographs of the specimen while measuring were taken (Figure 2a, 2b).

All the measurements were performed and recorded by two co-authors (TT and TK). The clamping force of the specimen by the caliper was defined as follows: the object does not fall during the measurement and leaves no caliper marks after the measuring process.

Postural assessment by X-ray imaging

**Figure 3:**
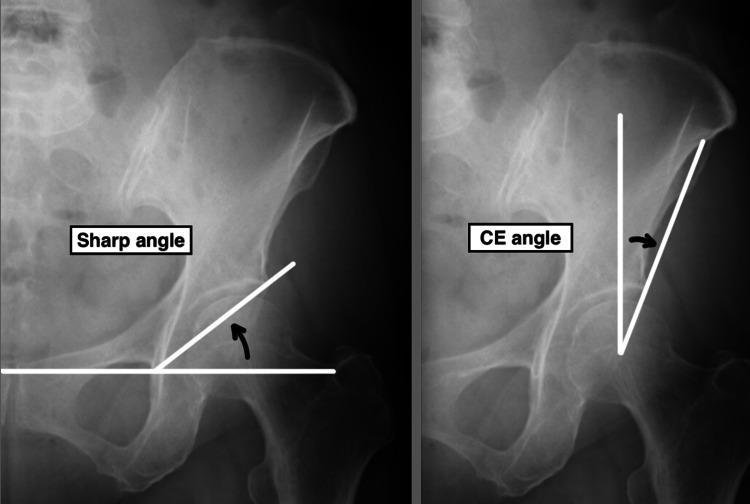
X-ray image of the hip joint, coronal plane CE: center-edge

**Figure 4:**
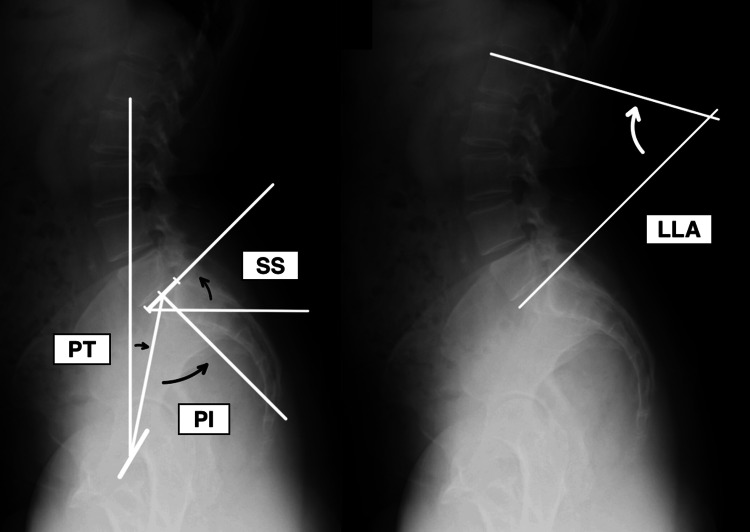
X-ray image of the hip joint while standing, sagittal plane SS: sacral slope; PT: pelvic tilt; PI: pelvic incidence; LLA: lumbar lordosis angle

For postural assessment by X-ray imaging, simple radiographs of the hip joint in the coronal plane and hip joint including the lumbar spine in the sagittal plane while standing were taken on day 1 before surgery. The Sharp angle and center-edge (CE) angle [25] and sacral slope (SS), pelvic tilt (PT), pelvic incidence (PI), and lumbar lordosis angle (LLA) [26] were measured by the main author (KY) three times (intraclass correlation coefficient (ICC)(1,1) >0.9; see Appendices) with each X-ray image by the use of DICOM viewer called X-Trek WebView system® (J-Mac System, Inc., Sapporo, Japan) (Figure 3 and Figure 4).

The definition of the radiographic angles is shown below. CE angle is the angle formed by the perpendicular line passing through the center of the femoral head and the line connecting the center of the femoral head and the outer edge of the acetabulum. The Sharp angle is the angle formed by the line connecting the lateral border of the acetabulum and the tip of the lacrimal root and the line connecting the tips of the bilateral lacrimal roots. Both angles are widely used as an indicator of DDH. SS is the angle between the superior border of the sacrum and the horizontal line, showing sacral tilt. PT is the angle between the vertical line from the midpoint connecting the center of the bilateral femoral heads and the line connecting the midpoint of the center of the bilateral femoral heads and the midpoint of the superior border of the sacrum. PT increases with posterior pelvic tilt and decreases with anterior pelvic tilt [27]. PI is the angle formed by two lines. One line is drawn from the midpoint of the centers of the bilateral femoral heads to the midpoint of the upper edge of the sacrum. The other line is a perpendicular line drawn from the midpoint of the upper edge of the sacrum. LLA is the angle formed by the line parallel to the upper border of the sacrum and the line parallel to the upper surface of the first lumbar vertebra. The severity of degeneration of hip OA was assessed from grades 1 to 3 by the Tönnis grade [28].

Passive ROM of the hip was measured to investigate the relationship with the pelvic alignment including flexion, extension, abduction, adduction, external rotation, and internal rotation. All the measurements were performed under the supervision of one of the co-authors (YM).

Basic attributes of the subjects

Basic attributes of the subjects (n=13) are shown in Table 1. 

**Table 1:** Basic attributes of the subjects

n=13 (female=13, male=0)	Mean±standard deviation
Age (years)	60.2(±8.57)
Height (cm)	156.4(±6.54)
Weight (kg)	59.1(±12.5)

Statistical analyses

For the statistical analysis, Spearman's correlation coefficient was examined between variables since the angular data from X-rays were used. To satisfy a correlation coefficient of r>0.5, a minimum of 26 cases were required with ES (r)=0.5, α=0.05, and 1-β=0.75. Principal component analysis was then applied to reduce the number of variables. R 4.1.3 (CRAN, freeware) was used for the analyses, with a significance level of 5%.

## Results

The main outcome

Table 2 shows the data of the main outcome including the Sharp angle, CE angle, SS, PT, PI, LLA, and the number of participants graded in the numerical rating of the Tönnis grade.

**Table 2:** The data of the main outcome †: the thickness of the capsule; CE: center-edge; SS: sacral slope; PT: pelvic tilt; PI: pelvic incidence; LLA: lumbar lordosis angle

Outcome		Mean±standard deviation
Thickness† (mm)		6.34(±1.72)
X-ray parameters (°)	Sharp angle	45(±5.45)
	CE angle	19.9(±12.04)
	SS	45.2(±8.14)
	PT	16.4(±5.38)
	PI	61.8(±8.49)
	LLA	53.8(±8.62)
Tönnis grade (1-3)	Grade 1	2 cases
	Grade 2	6 cases
	Grade 3	5 cases

The secondary outcome

Hip ROM as the secondary outcome was summarized in Table 3.

**Table 3:** The data of the secondary outcome ROM: range of motion

Outcome		Mean±standard deviation
ROM (°)	Flexion	78.5(±17.72)
	Extension	-2.7(±8.57)
	Abduction	21.5(±11.25)
	Adduction	10.8(±5.72)
	External rotation	20.8(±14.27)
	Internal rotation	7.3(±9.71)

Correlation coefficient for each variable

The correlation matrix for each variable is shown in Table 4. The p-value of each correlation coefficient is shown in Table 5. A significant negative correlation was found between capsule thickness and the Sharp angle (r=-0.57, p<0.05). Based on this correlation coefficient, the power was determined to be 0.57 (57%). No significant correlation was found between SS, PT, PI, and LLA and hip joint capsule thickness.

**Table 4:** The correlation matrix for each variable †: capsule thickness; ‡: CE angle; §: Sharp angle; CE: center-edge; SS: sacral slope; PT: pelvic tilt; PI: pelvic incidence; LLA: lumbar lordosis angle

	CEA‡	LLA	PI	PT	SS	SharpA§
Thickness†	0.121	-0.187	-0.006	-0.212	-0.080	-0.568
CEA‡	1.000	0.073	0.021	0.023	-0.094	-0.349
LLA		1.000	0.386	0.004	0.701	-0.198
PI			1.000	0.628	0.758	-0.166
PT				1.000	0.120	0.248
SS					1.000	-0.325

**Table 5:** The p-value for each correlation coefficient †: capsule thickness; ‡: CE angle; §: Sharp angle; CE: center-edge; SS: sacral slope; PT: pelvic tilt; PI: pelvic incidence; LLA: lumbar lordosis angle

	CEA‡	LLA	PI	PT	SS	SharpA§
Thickness†	0.693	0.541	0.986	0.487	0.796	0.043
CEA‡	0.000	0.812	0.947	0.939	0.761	0.242
LLA		0.000	0.193	0.989	0.008	0.517
PI			0.000	0.022	0.003	0.588
PT				0.000	0.697	0.413
SS					0.000	0.279

Principal component analysis

The result of the principal component analysis is shown in Table 6. The results were obtained up to the second principal component, showing that the first principal component was strongly related to LLA, PI, and SS, while the second principal component was strongly related to hip joint capsule thickness, CE angle, and Sharp angle.

**Table 6:** The principal component analysis PC: primary component; CE: center-edge; SS: sacral slope; PT: pelvic tilt; PI: pelvic incidence; LLA: lumbar lordosis angle

Variable	PC1	PC2
Capsule thickness	0.276	0.784
CE angle	0.127	0.755
Sharp angle	0.184	-0.884
LLA	-0.710	0.069
PI	-0.892	0.068
SS	-0.910	0.130
PT	-0.449	-0.176
Percentage％	35.10%	28.89%
Cumulative％	35.10%	63.99%

## Discussion

The results of this study showed a negative correlation between the Sharp angle of the X-ray index on the coronal plane and the thickness of the anterior hip capsule. All subjects had acetabular dysplasia in the present study, and the Tönnis grade in the subjects of this study is biased toward grade 3. In addition, taking into account the results of the hip ROM with the participants of the present study, the tendency was indicated that the participants of the present study were toward severe degeneration of the hip joint. Although the hip capsule may thicken in the early stage of hip pain [13,14], it is still unknown whether the capsule thickness changes with the progression of OA. Kijima et al. suggested that the pain subsided with the forming of roof osteophyte [29], which can explain why bony structure plays a more important role in the stability of the joint in the later degenerative process of DDH.

Meanwhile, no significant correlation was found between anterior hip capsule thickness and CE angle, SS, PT, PI, and LLA. The correlation coefficients for X-ray indices were higher in the order of Sharp angle, PT, LLA, CE angle, SS, and PI. Between them, only PT and Sharp angle showed low to moderate negative correlation coefficients. This result indicates that the pelvic alignment in the coronal plane and the sagittal plane for DDH in our population may be related. PI had the lowest correlation coefficient which can be explained because PI is the pelvic angle unique to each individual in terms of morphology. The normal angles in Japan were shown below: Sharp angle: 41.5±3.5, CE angle: 27.8±6.8, SS: 35.9±9.9, PT: 16.9±5.8, PI: 51.9±10.4, and LLA: 51.3±12.8 [26,30]. In comparison with the result of the present study, the Sharp angle was larger and the CE angle was smaller than in the healthy population which explains why the diagnosis of DDH for the participants of the present study is reasonable. SS and PI in the present study were larger. On the other hand, PT and LLA were similar to the healthy population. Though the correlation coefficient was low to moderate in PT and Sharp angle in the present study, the angle of PT was in the range of the normal population. This may indicate that the angle of PT is not relevant to DDH in our population. Careful interpretation of the result is required because all the correlations except for the Sharp angle were not significant and the normal range of each X-ray index varies between countries and age groups [1,2,25,30,31]. At least, the present study revealed the correlation coefficient between anterior hip capsule thickness and Sharp angle; the hypothesis of this study about a relationship between anterior hip capsule thickness and pelvic alignment can be further verified if a larger number of subjects are examined in the future.

The results of the principal component analysis showed that anterior hip capsule thickness, CE angle, and Sharp angle tended to be strongly related to the second principal component. The interpretation of the first principal component was applied as sagittal radiographic indices because LLA, PI, and SS were related as suggested by previous studies [20,21]. The second principal component was interpreted as the degree of joint instability in the hip. The reason can be explained that the thickness of the anterior hip capsule may be more influenced by the coronal plane than the sagittal plane of the X-ray index. Further validation of the relationship between the variables is needed.

Regarding the anterior hip capsule thickness, the results of this study showed different values from the previous reports. Devitt et al. measured the hip capsule thickness by using arthroscopy in young patients with hip pain and reported the thickness as 7.5 mm on average [13] at the location of 2 o'clock position on the clock face as defined by Philippon et al. [32], which is thicker than average for the subjects in this study. On the other hand, a cadaveric study measured by Walters et al. without deformity of the hip joint showed the capsule thickness as 3.6 mm on average in the anterosuperior area, which was clearly thinner than the present study [12]. The location of measurement in the present research was estimated to be the 12 to 3 o'clock position on the clock face as defined by Philippon et al. [32]; therefore, it can be said that they are similar in terms of measurement locations. However, the setting of the research varied in terms of the disease stage, in vivo or in vitro, as well as genders and races of participants. The future study will be encouraged to measure the validity and reliability of each measurement for anterior capsule thickness by using standardized methods of measuring including measuring the location of the anterior hip capsule based on multi-centered research within the same and/or multiple areas, because the geographical and gender differences and ethnic factors including the lifestyle of DDH patients may be associated with capsule thickness [1,2,4,5,25,30].

The specimen in the present study consisted of the thickened area of the lateral fiber of the iliofemoral ligament based on its anatomical location of surgery. It is worth noting that the medial side of the specimen may partially contain the lateral end of the medial fiber of the iliofemoral ligament. Additionally, on the lateral side, there is a relatively thin area of the articular capsule. The pubofemoral ligament is located more medially, and it is out of range of the specimen [24]. Short et al. proposed that the femoral nerve is responsible for innervating the superior lateral side of the hip capsule, which mainly consists of the lateral fiber of the iliofemoral ligament. On the other hand, the obturator nerve is responsible for innervating the area located on the inferior lateral side of the hip joint capsule. It is important to note that these nerves are located in the peripheral region and have only the sensory branch [33].

The advantage of the method of this study is the use of direct measurement with the fresh specimen as close as possible to an in vivo sample, in the surgery of THA. Furthermore, the direct measurement is technically easy because the specimen was simply placed between the calipers, measured, photographed, and recorded. Therefore, it is more cost-effective than the other styles of measurements of the hip capsule by the previous studies and also free of ethical issues. On the other hand, this method is inferior to MRI regarding the timing of the intervention as it can only be approached when THA is performed. Moreover, the boundary between the muscle and joint capsule is more precise in MRI than the direct measurement. Conversely, on MRI, the composition of the capsule tissue itself cannot be assessed. A cadaveric study suggests that the boundary between the articular capsule tissue and the muscular tissue such as the iliocapsularis muscle is vague because the direct attachment with these tissues in the capsule was observed [29]. Thus, the investigation of the composition of the hip capsule of DDH should be expected in the future. A wider range of patients with hip arthritis can be evaluated if the appropriate timing of measurement of the hip capsule was performed in the degenerating process of DDH.

As a limitation of this study, the small number of subjects is considered to be the one that most influenced the results. The correlation coefficient between capsular thickness and Sharp angle, which showed a significant correlation, did not reach the target power of 0.75, but a comparatively high value of 0.57 was shown. Reliability could be increased by increasing the number of subjects. The correlation coefficient between anterior hip capsule thickness and Sharp angle was moderately high, but it is likely to be lower as the number of subjects increases. The interpretation of the trends based on the results of principal component analysis is also likely to change as the number of subjects increases. Second, fatty tissue was removed as much as possible from the excised hip capsule; however, the composition of the capsule is currently unknown because pathological diagnosis and other histological verifications have not been performed. Moreover, some conditions including that the data was collected at the same clinic without blinding, the inconsideration of the severity of hip degeneration and morphological characteristics such as height and weight, the small number of THA cases in the research period, and the male-female ratio of DDH which tends to be more common among women in the identical geographical location may have influenced the result. Particularly, patients with hip OA as a result of DDH may be associated with non-symptomatic lumbar spondylolisthesis, which may have affected their posture including pelvic alignment and lumbar lordosis. Future studies should include these factors in the inclusion criteria for a better understanding of the relationship between hip capsule thickness and pelvic alignment and lumbar lordosis among DDH patients.

## Conclusions

A negative correlation was found between the thickness of the hip capsule and the Sharp angle of the radiographic index on the anterior plane in patients undergoing THA due to the diagnosis of DDH. On the other hand, no significant correlation was obtained between hip capsular thickness and the sagittal plane X-ray indices such as SS, PT, and PI. The results showed no clear relationship between anterior capsule thickness and pelvic alignment in patients with DDH. However, the results of this study suggest that the thickness of the joint capsule does not necessarily relate to the degenerative process among patients with DDH and the process can be complex to apply two-dimensional postural indices for the explanation. Further consideration of the hip capsule thickness in DDH may help to better understand the pathology of DDH.

## Appendices

**Table 7:** The intra-examiner reliability, ICC(1,1) for the measurement of X-ray indices ICC: intraclass correlation coefficient; CE: center-edge; SS: sacral slope; PT: pelvic tilt; PI: pelvic incidence; LLA: lumbar lordosis angle

	ICC	95%lower	95%upper
Sharp angle	0.927	0.830	0.975
CE angle	0.982	0.957	0.994
SS	0.960	0.904	0.986
PT	0.977	0.944	0.992
PI	0.964	0.913	0.988
LLA	0.961	0.906	0.987
